# First-Line Tyrosine Kinase Inhibitors Combined With Local Consolidative Radiation Therapy for Elderly Patients With Oligometastatic Non-Small Cell Lung Cancer Harboring EGFR Activating Mutations

**DOI:** 10.3389/fonc.2022.766066

**Published:** 2022-01-25

**Authors:** Xiaolong Hu, Hongqi Li, Xiaoli Kang, Xuan Wang, Haifeng Pang, Chen Liu, Jianchun Zhang, Yingjie Wang

**Affiliations:** ^1^Department of Radiation Oncology, Beijing Geriatric Hospital, Beijing, China; ^2^Department of Radiation Oncology, Air Force General Hospital, Beijing, China

**Keywords:** EGFR mutant NSCLC, local consolidation radiation, residual disease, oligometastatic, elderly patients

## Abstract

**Objective:**

The aim of this study was to investigate the efficacy and safety of combined applications of local consolidative radiation therapy (LCRT) and first-line tyrosine kinase inhibitors (TKIs) for the treatment of primary tumors and oligometastatic sites in oligometastatic NSCLC harboring Epidermal Growth Factor Receptor (EGFR) activating mutations.

**Patients and Methods:**

Elderly patients with oligometastatic NSCLC (≤5 metastases) harboring EGFR activating mutations at the time of diagnosis were identified. They were treated with first-line TKIs alone or in combination with LCRT. Progression‐free survival (PFS) and overall survival (OS) were estimated through the Kaplan–Meier method.

**Results:**

A total of 122 elderly patients were enrolled between February 2010 and January 2018. Among them, 41.0% (n = 50) received TKIs combined with LCRT (TKIs + LCRT group), whereas 59.0% (n = 72) received TKIs monotherapy (TKIs alone group). Patients were followed up for a median length of 34 months (ranging from 7.0 to 64 months). The median PFS in TKIs + LCRT group was 17 months (95%CI: 15.37–18.63), which was significantly longer than that of the TKIs-alone group (12 months; 95%CI: 11.05–12.95) (p <0.001). Median OS in TKIs + LCRT group was 38 months (95%CI: 35.61–40.39), while that of the TKIs-alone group was 29 months (95%CI: 26.86–31.14) (p <0.001). Multivariate analyses revealed that LCRT, one to two metastases, and good ECOG PS were independent predictors for better PFS (p <0.001, p = 0.004, and p = 0.027). Moreover, LCRT, good ECOG PS, and T_1-2_ stage were independent predictors for better OS (p <0.001, p = 0.007 and p = 0.007). Most of the patients suffered from grade 1 to 2 toxicities, and treatment-related deaths were not recorded.

**Conclusion:**

First-line TKIs combined with LCRT may improve survival outcomes for elderly patients with oligometastatic NSCLC harboring EGFR activating mutations. This approach was not associated with much toxicity, therefore, it can be used for the treatment of elderly patients with oligometastatic disease.

## Introduction

The prevalence of non-small cell lung cancer (NSCLC) among elderly people has been increasing. Approximately 50% of NSCLC patients present with distant metastases at the time of diagnosis (1). In advanced NSCLC patients with sensitizing EGFR mutations, EGFR-TKIs have been found to effectively improve clinical outcomes, relative to cytotoxic chemotherapies (2–5). However, within 9–12 months of treatment, most patients develop resistance to EGFR-TKIs (6, 7). In 1995, Hellman proposed the concept of “oligometastasis”, which is the transition stage between local primary tumors and extensive metastasis. Several randomized clinical trials have evaluated the efficacy of different treatments for patients with oligometastatic NSCLC (8–10). However, available treatments are not effective for geriatric patients with NSCLC harboring EGFR mutations, without T790M-mediated resistance to their initial EGFR inhibitor. Unlike younger patients, elderly patients are often administered with less aggressive treatments, possibly due to resistance to EGFR-TKIs. Therefore, there is a need to develop treatment options that can suppress tumor progression among elderly patients. A combination of local consolidation therapy and EGFR-TKIs in patients with oligometastatic NSCLC harboring EGFR activating mutations significantly prolonged PFS and overall survival (OS), relative to TKIs alone (11). Currently, the efficacy and safety of combined local consolidative radiation therapy (LCRT) and first-line EGFR-TKIs in elderly patients with oligometastatic NSCLC harboring EGFR activating mutations have not been clearly defined. In this study, we tested the hypothesis that elderly patients with oligometastatic NSCLC harboring EGFR mutations may benefit from combined treatments of LCRT and first-line EGFR-TKIs.

## Materials and Methods

### Patients

Stage IV EGFR-mutant NSCLC patients with oligometastatic disease within 1 month of diagnosis at the Beijing Geriatric Hospital and Air Force General Hospital were retrospectively analyzed between January, 2010 and February, 2018. The inclusion criteria were: 65 years or older (elderly), pathologically confirmed NSCLC harboring EGFR-sensitizing mutations, patients with synchronous oligometastatic disease (≤5 metastases) confirmed by comprehensive imaging examinations (namely, brain magnetic resonance imaging (MRI) + whole-body positron emission tomography computed tomography (PET-CT) or brain MRI + thoracic/abdominal/pelvic CT, and bone scan when necessary), Eastern Cooperative Oncology Group Performance Status (ECOG PS) score of 2 or less, after initial treatment with first-line EGFR-TKIs, the primary tumor and all metastases were stable.

Treatment responses were assessed 6–8 weeks after treatment with first-line TKIs, in accordance with response evaluation criteria for solid tumors, Revised RECIST guidelines (version 1.1). The EGFR-TKIs used in this study were gefitinib (250 mg daily) and erlotinib (150 mg daily). Patients that responded well to first-line TKIs followed by treatment with local consolidative radiation therapy (LCRT) to primary tumor and all oligometastatic sites were designated as the TKIs + LCRT group. Patients treated with first-line TKIs alone were designated as the TKIs-alone group. Progression‐free survival was defined as the time from initiation of TKIs treatment to progression or death from any cause. Overall survival was defined as the duration between the date of TKIs initiation and date of death.

### Local Consolidative Radiation Therapy Procedure

The application of local consolidative radiation therapy was decided by a multidisciplinary team (namely, oncologists, radiation oncologists, radiologists, orthopedic surgeons, neurosurgeons, cardiologists, respiratory physicians, and geriatricians) at each center. Clinical assessment included the evaluation of factors such as age, cardiopulmonary functions, underlying diseases, pathological fracture risk, nutritional status, central nervous system symptoms, and risk–benefit ratio. The local consolidative radiation therapy regimen was determined by radiation oncologists based on general conditions, tumor locations, tumor sizes, tumor boundary, pulmonary functions, bone marrow, hepatic, and renal functions.

The primary tumor was subjected to hypofractionated radiotherapy (70 Gy in 10–15 fractions, the biologically effective dose was 103–119 Gy) or conventional fractionated radiotherapy (70 Gy in 30–35 fractions, the biologically effective dose was 84.0–86.3 Gy). Vertebral metastases were treated with conventional fractionated radiotherapy (30 Gy in 10 fractions or 40 Gy in 20 fractions), isolated brain metastases were treated with gamma knife radiosurgery (50% Isodose line 22 Gy/1f), multiple brain metastases were treated with conventional fractionated radiotherapy (60 Gy in 20 fractions to brain metastases) or conventional fractionated radiotherapy plus whole-brain radiation therapy (50 Gy in 20 fractions to brain metastases plus WBRT 40 Gy in 20 fractions). Liver metastases were managed *via* hypofractionated radiotherapy (65 Gy in 20 fractions or 60 Gy in 15 fractions). Contralateral lung metastases were treated with gamma knife radiosurgery (70% Isodose line70–78 Gy/10–14 f), hypofractionated radiotherapy (70 Gy in 15 fractions or 60 Gy in 15 fractions) or conventional fractionated radiotherapy (60 Gy in 20 fractions). Non-regional lymph node metastases were controlled by conventional fractionated radiotherapy (70 Gy in 30–35 fractions or 60 Gy in 30 fractions).

### Toxicity Assessment

Acute and long-term toxicities were defined before and after 90 days using the Common Terminology Criteria for Adverse Events (CTCAE) version 5 in accordance with the National Cancer Institute (NCI). TKIs-related acute toxicities included skin rash, diarrhea, pneumonitis, neutropenia, fatigue, vomiting, and elevated ALT levels. Radiotherapy-related acute toxicities included pneumonia, fatigue, thrombocytopenia, anemia, leukopenia, dermatitis, and esophagitis.

### Statistical Analysis

Statistical analyses were performed using the IBM Statistical Package for Social Sciences (SPSS) software 25 (SPSS Inc., Chicago, IL, USA), and GraphPad Prism 7 version 7.04 (GraphPad Software, Inc., La Jolla, California USA). Normally distributed quantitative data are expressed as mean and standard deviation (M ± SD). Categorical variables are presented in percentages and frequency distributions. Kaplan–Meier analysis with the log-rank test was used to calculate and analyze survival curves. A Cox proportional hazards model was used for univariate and multivariate analyses to assess possible prognostic factors and calculate survival hazard ratios (HRs) for PFS and OS at 95% confidence intervals (95%CI). The significant parameters identified in univariate analyses (p <0.05) were incorporated into multivariate Cox regression analysis to determine the independent prognostic factors. A two-sided p <0.05 was considered statistically significant.

## Results

### Patient Characteristics

**Figure 1:**
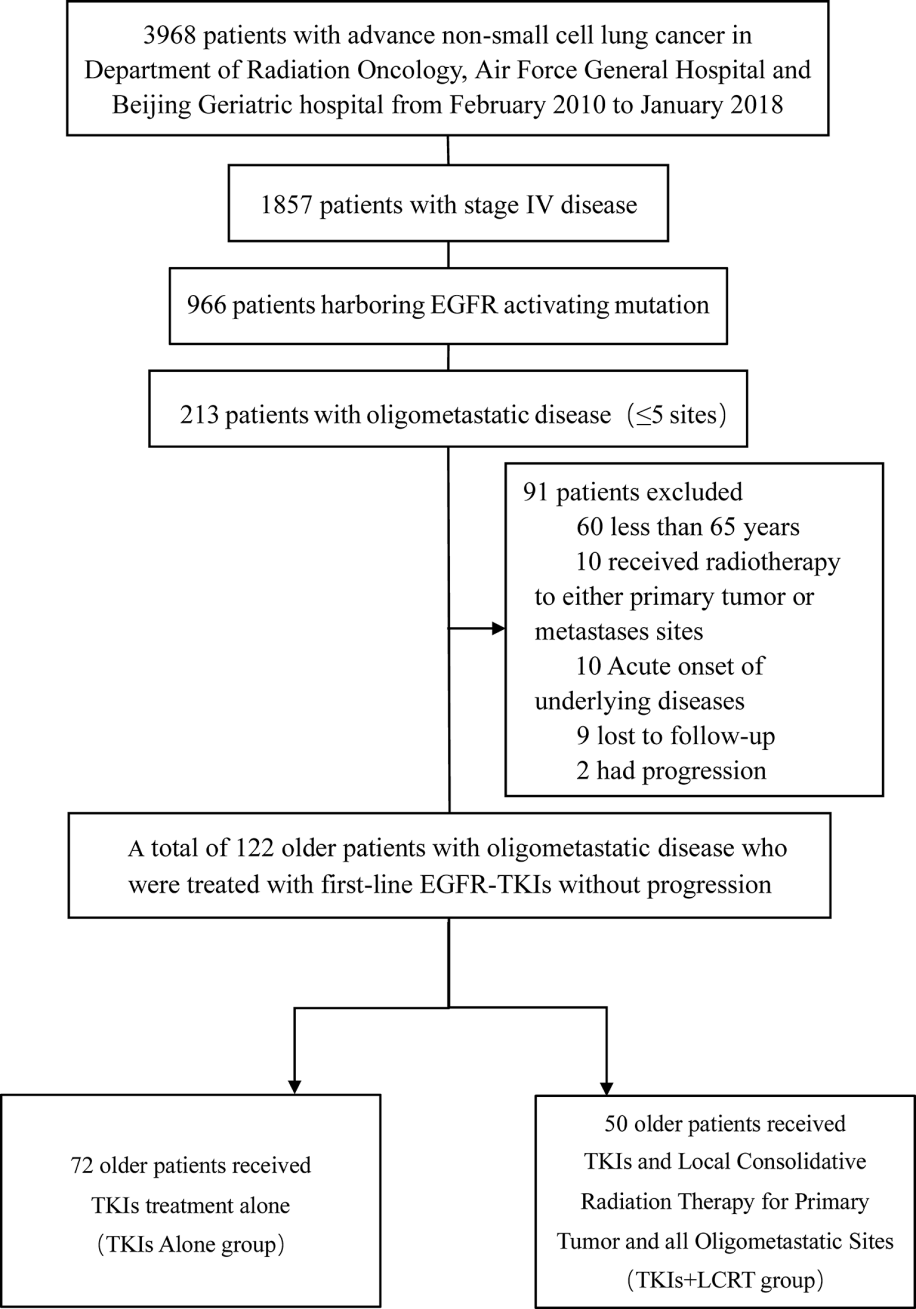
Flowchart showing selection process for patients. EGFR, Epidermal Growth Factor Receptor; TKIs, Tyrosine Kinase Inhibitors; LCRT, Local Consolidative Radiation Therapy.

A total of 122 patients with oligometastatic NSCLC (≤5 metastases) harboring the EGFR sensitizing mutation and treated with first line TKIs without progression were enrolled between February 2010 and January 2018 (**Figure 1**). **Table 1** shows the general clinical characteristics for the enrolled patients. The patients had a median age of 72.5 years, with 39.3% (n = 48) of them being older than 75 years. Majority of the patients (n = 102, 83.6%) had adenocarcinoma histology, 4.9% (n = 6) had squamous cell carcinoma, 54.9% (n = 67) had Exon 21 L858R mutations, 59% (n = 72) were present or former smokers, 80.3% (n = 98) had underlying diseases, 63.9% (n = 78) had hypertension, 39.3% (n = 48) had diabetes, 28.7% (n = 35) had chronic obstructive pulmonary disease (COPD), 52.5% (n = 64) had ECOG PS 0 or 1, 46.7% (n = 57) had N_0–1_ stage, while 51.6% (n = 63) had T_1–2_ stage. Most of the patients (77.9%, n = 95) received gefitinib as first-line treatment. Regarding the quantity of oligometastatic lesions in the 122 patients, 55.7% (n = 68) had one to two metastases, with 8 patients having the brain as the only site for metastases. Moreover, 44.3% (n = 54) of the patients had three to five metastases. With respect to oligometastatic sites, 50.8% (n = 62) patients had brain metastases, 48.3% (n = 59) had bone metastases, 34.4% (n = 42) had contralateral lung metastases, 28.7% (n = 35) had adrenal metastases, 7.4% (n = 9) had liver metastases, while 6.60% (n = 8) had non-regional lymph nodes metastases.

**Table 1:** Baseline patient characteristics.

Characteristics	N	No. of Patients (%)
Gender		
Male	41	33.6%
Female	81	66.4%
Age		
65–75	74	60.7%
>75	48	39.3%
EOCG performance status		
0–1	64	52.5%
2	58	47.5%
Histology		
Adenocarcinoma	102	83.6%
Adenosquamous	9	7.4%
Squamous cell	6	4.9%
NSCLC	5	4.1%
EGFR mutation		
Exon 19 deletion	55	45.1%
Exon 21 L858R	67	54.9%
Smoking status		
Nonsmoker	50	41.0%
Present or former smoker	72	59.0%
Smoking Index		
<600	71	58.2%
≥600	51	41.8%
Comorbidity		
No	24	19.7%
Yes	98	80.3%
Comorbidity type		
Hypertension	78	63.9%
Diabetes	48	39.3%
COPD	35	28.7%
CHD	30	24.6%
Atrial Fibrillation	21	17.2%
N stage		
N0–1	57	46.7%
N2–3	65	53.3%
T stage		
T1–2	63	51.6%
T3–4	59	48.4%
Second-line treatment		
No	58	47.5%
Yes	64	52.5%
No. of metastases		
1–2	68	55.7%
3–5	54	44.3%
Metastasis location		
Brain	62	50.8%
Bone	59	48.3%
Lung	42	34.4%
Adrenal	35	28.7%
Liver	9	7.40%
Non-region lymph nodes	8	6.60%
PET-CT		
No	37	30.3%
Yes	85	69.7%
LCRT for both PT and OS		
No	72	59.0%
Yes	50	41.0%

NSCLC, non-small cell lung cancer; Smoking Index, number of cigarettes smoked per day × years of smoking; ECOG PS, Eastern Cooperative Oncology Group Performance Status; COPD, Chronic Obstructive Pulmonary Disease; CHD, Coronary Heart Disease; CNS, Central Nervous System; BED, Biological Effective Dose; PET-CT, Positron Emission Tomography Computed Tomography; PT, Primary Tumor; OS, Oligometastatic Sites; LCRT, Local Consolidative Radiation Therapy; EGFR, Epidermal Growth Factor Receptor.

Overall, 41% (n = 50) of the patients received local consolidative radiation therapy to primary tumor and all oligometastatic sites (**Table 2**). Thirty two patients in the TKIS + LCRT group received hypofractionated radiotherapy (BED_10_ ≥100 Gy) for the primary tumor (**Table 3**), 4 patients received gamma knife radiosurgery for isolated brain metastases, 13 patients received conventional radiotherapy plus whole-brain radiation therapy, 8 patients received gamma knife radiosurgery for contralateral lung metastases, 4 patients received hypofractionated radiotherapy for adrenal metastases, while 2 patients received hypofractionated radiotherapy for liver metastases.

**Table 2:** Patients characteristics of tkis alone group and LCRT + TKIs group.

Characteristics	TKIs Alone (n = 72)	LCRT + TKIs (n = 50)
	No. (%)	No. (%)
Gender		
Male	24 (33.3%)	17 (34.0%)
Female	48 (66.7%)	33 (66.0%)
Age		
65–75	47 (65.3%)	27 (54.0%)
>75	25 (34.7%)	23 (46.0%)
EOCG performance status		
0–1	39 (54.2%)	25 (50.0%)
2	33 (45.8%)	25 (50.0%)
Histology		
Adenocarcinoma	72 (80.6%)	44 (88.0%)
Nonadenocarcinoma	14 (19.4%)	6 (12.0%)
EGFR mutation		
Exon 19 deletion	32 (44.4%)	23 (46.0%)
Exon 21 L858R	40 (55.6%)	27 (54.0%)
Smoking status		
Nonsmoker	34 (47.2%)	16 (32.0%)
Present or former smoker	38 (52.8%)	34 (68.0%)
Smoking Index		
<600	47 (65.3%)	24 (48.0%)
≥600	25 (34.7%)	26 (52.0%)
Comorbidity		
No	31 (43.1%)	19 (38.0%)
Yes	41 (56.9%)	31 (62.0%)
N stage		
N0–1	38 (52.8%)	19 (38.0%)
N2–3	34 (47.2%)	31 (62.0%)
T stage		
T1–2	41 (56.9%)	22 (44.0%)
T3–4	31 (43.1%)	28 (56.0%)
Second-line treatment		
No	34 (47.2%)	24 (48.0%)
Yes	38 (52.8%)	26 (52.0%)
No. of metastases		
1–2	38 (52.8%)	30 (60.0%)
3–5	34 (47.2%)	20 (40.0%)
**Metastasis location**		
Brain		
No	36 (50%)	24 (48.0%)
Yes	36 (50%)	26 (52.0%)
Bone		
No	32 (44.4%)	31 (62.0%)
Yes	40 (55.6%)	19 (38.0%)
Lung		
No	44 (61.1%)	36 (72.0%)
Yes	28 (38.9%)	14 (28.0%)
Adrenal		
No	49 (68.1%)	38 (76.0%)
Yes	23 (31.9%)	12 (24.0%)
Liver		
No	66 (91.7%)	47 (94.0%)
Yes	6 (8.3%)	3 (6.0%)
Non-region lymph nodes		
No	66 (91.7%)	48 (96.0%)
Yes	6 (8.3%)	2 (4.0%)
PET-CT		
No	25 (34.7%)	12 (24.0%)
Yes	47 (65.3%)	38 (76.0%)

NSCLC, non-small cell lung cancer; Smoking Index, number of cigarettes smoked per day × years of smoking; ECOG PS, Eastern Cooperative Oncology Group Performance Status; TKIs, Tyrosine Kinase Inhibitors; EGFR, Epidermal Growth Factor Receptor; PET-CT, Positron Emission Tomography Computed Tomography; LCRT, Local Consolidative Radiation Therapy.

**Table 3:** Local radiation therapy for primary tumor and oligometastatic sites mode.

Sites of Disease/Treatment Regimen	Patients	No. of Patients (%)
Primary tumor and region lymph nodes	50	
Dt 70 Gy/10 f	10	20.0%
Dt 70 Gy/15 f	22	44.0%
Dt 70 Gy/30–35 f	18	36.0%
**Metastasis location**		
Brain	26	
Dt 50% Isodose line 22 Gy/1 f^*^	4	15.4%
Dt 60 Gy/20 f	9	34.6%
Dt 50 Gy/20 f + WBRT	13	50.0%
Bone	19	
Dt 30 Gy/10 f	10	52.6.%
Dt 40 Gy/20 f	9	47.4%
Lung	14	
Dt 70% Isodose line70–78 Gy/10–14 f^#^	8	57.2%
Dt 70 Gy/15 f	1	7.1%
Dt 60 Gy/15 f	2	14.3%
Dt 60 Gy/20 f	3	21.4%
Adrenal	12	
Dt 70 Gy/10–15 f	4	33.3%
Dt 60 Gy/20 f	7	58.3%
Dt 45 Gy/15 f	1	8.40%
Liver	3	
Dt 65 Gy/20 f	1	33.3%
Dt 60 Gy/15 f	2	66.7%
Non-region lymph nodes	2	
Dt 70 Gy/30–35 f	1	50.0%
Dt 60 Gy/30 f	1	50.0%

Dt, Dose of Target; WBRT, Whole Brain Radiation Therapy.

^*^The Head Gamma Knife.

^#^The Body Gamma Knife.

### Survival Outcomes

**Figure 2:**
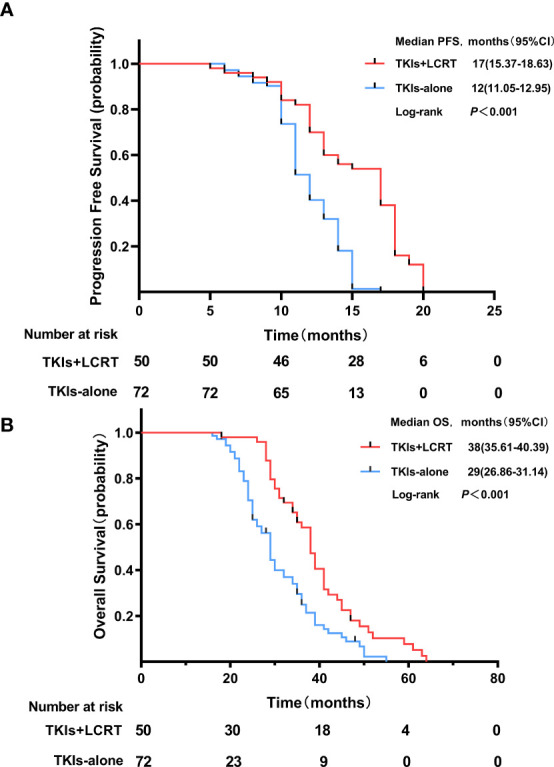
Progression free survival **(A)** and overall survival **(B)** of patients treated with TKIs + LCRT or TKIs alone for oligometastatic NSCLC harboring EGFR activating mutations.

The median length of follow-up time was 34 months (range, 7.0–64 months). The median progression free survival (mPFS) time in the TKIs + LCRT group was 17 months, while that of the TKIs-alone group was 12 months (p <0.001). The median overall survival (mOS) time in the TKIs + LCRT group was 38 months, while that of the TKIs-alone group was 29 months (p <0.001) (**Figure 2**). The mPFS in the entire study population was 13 months, while mOS was 34 months (95%CI: 30.3–37.7).

### Univariate and Multivariate Analyses of PFS and OS

Univariate analysis revealed that LCRT for primary tumor and all oligometastatic sites resulted in better PFS (HR = 0.30, p <0.001). A similar observation was made for one to two metastases (HR = 0.49, p <0.001), and good ECOG PS (HR = 0.68, p = 0.035). Multivariate analysis revealed that LCRT for primary tumor and all oligometastatic sites was an independent predictive factor for better PFS (HR = 0.32, 95%CI: 0.20–0.51, p <0.001). This was the case for one to two metastases (HR = 0.57, 95%CI: 0.39–0.83, p = 0.004), and good ECOG PS (HR = 0.67, 95%CI: 0.46–0.96, p = 0.027) (**Table 4**).

**Table 4:** Factors associated with progress free survival in univariate and multivariate analyses.

Variable	Univariable			Multivariable		
	HR	95%CI	*P*	HR	95%CI	*P*
Gender						
Male vs. Female	0.97	0.67–1.41	0.871			
Age						
65–75 vs. >75	0.78	0.55–1.14	0.792			
EOCG performance status						
0–1 vs. 2	0.68	0.47–0.97	***0.035***	0.67	0.46–0.96	***0.027***
Histology						
Adenocarcinoma vs. Nonadenocarcinoma	0.93	0.57–1.49	0.750			
EGFR mutation						
Exon 19 deletion vs. Exon 21 L858R	1.05	0.74–1.51	0.776			
Smoking status						
Present or former smoker vs. Nonsmoker	1.09	0.75–1.55	0.682			
Smoking Index						
≥600 vs.<600	1.02	0.71–1.46	0.924			
Comorbidity						
Yes vs. No	1.16	0.81–1.67	0.413			
T stage						
T1–2 vs. T3–4	0.84	0.59–1.21	0.348			
N stage						
N0–1 vs. N2–3	0.97	0.68–1.40	0.900			
CNS metastases						
Yes vs. No	1.10	0.77–1.56	0.623			
No. of metastases						
1–2 vs. 3–5	0.49	0.33–0.71	***<0.001***	0.57	0.39–0.83	***0.004***
LCRT for both PT and OS						
Yes vs. No	0.30	0.19–0.48	***<0.001***	0.32	0.20–0.51	***<0.001***

NSCLC, non-small cell lung cancer; Smoking Index, number of cigarettes smoked per day × years of smoking; ECOG PS, Eastern Cooperative Oncology Group Performance Status; CNS, Central Nervous System; EGFR, Epidermal Growth Factor Receptor; PT, Primary Tumor; OS, Oligometastatic Sites; LCRT, Local Consolidative Radiation Therapy.

The bold values indicate significant P values.

**Figure 3:**
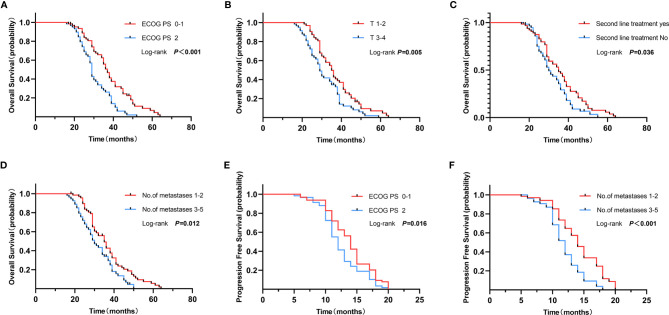
Overall survival of ECOG PS 0–1 vs. 2 **(A)**, T_1–2_ vs. T_3–4_ **(B)**, Second line treatment yes vs. no **(C)** and no. of metastases 1–2 vs. 3–5 **(D)**. Progression free survival of ECOG PS 0–1 vs. 2 **(E)**, and no. of metastases 1–2 vs. 3–5 **(F)**.

Univariate analysis showed that patients who received LCRT to primary tumor and all oligometastatic sites were associated with better OS (HR = 0.48, p <0.001), T_1–2_ stage (HR = 0.60, p = 0.008), good ECOG PS (HR = 0.46, p <0.001), second-line treatment (HR = 0.67, p = 0.045), and presented with one to two metastases (HR = 0.62, p = 0.016). Multivariate analysis showed that patients that received LCRT to primary tumor and all oligometastatic sites was an independent prognostic factor for better OS (HR = 0.41, 95%CI: 0.27–0.63, p <0.001), good ECOG PS (HR = 0.54, 95%CI: 0.34–0.85, p = 0.007), and T_1–2_ stage (HR = 0.56, 95%CI: 0.37–0.85, p = 0.007) (**Table 5** and **Figure 3**).

**Table 5:** Factors associated with overall survival in univariate and multivariate analyses.

Variable	Univariable			Multivariable		
	HR	95%CI	*P*	HR	95%CI	*P*
Gender						
Male vs. Female	0.90	0.61–1.34	0.612			
Age						
65–75 vs. >75	0.79	0.53–1.16	0.224			
EOCG performance status						
0–1 vs. 2	0.46	0.33–0.72	***<0.001***	0.54	0.34–0.85	***0.007***
Histology						
Adenocarcinoma vs. Nonadenocarcinoma	0.88	0.52–1.48	0.620			
EGFR mutation						
Exon 19 deletion vs. Exon 21 L858R	0.99	0.68–1.46	0.999			
Smoking status						
Present or former smoker vs. Nonsmoker	1.38	0.93–2.02	0.115			
Smoking Index						
≥600 vs.<600	1.06	0.72–1.55	0.777			
Second-line treatment						
Yes vs. No	0.67	0.45–0.99	***0.045***	0.95	0.63–1.46	0.833
Comorbidity						
Yes vs. No	1.23	0.83–1.82	0.300			
T stage						
T1–2 vs. T3–4	0.60	0.41–0.87	***0.008***	0.56	0.37–0.85	***0.007***
N stage						
N0–1 vs. N2–3	0.80	0.54–1.18	0.258			
CNS metastases						
Yes vs. No	1.20	0.82–1.76	0.342			
No. of metastases						
1–2 vs. 3–5	0.62	0.42–0.92	***0.016***	0.86	0.57–1.29	0.458
LCRT for both PT and OS						
Yes vs. No	0.48	0.32–0.72	***<0.001***	0.41	0.27–0.63	***<0.001***

NSCLC, non-small cell lung cancer; Smoking Index, number of cigarettes smoked per day × years of smoking; ECOG PS, Eastern Cooperative Oncology Group Performance Status; CNS, Central Nervous System; EGFR, Epidermal Growth Factor Receptor; PT, Primary Tumor; OS, Oligometastatic Sites; LCRT, Local Consolidative Radiation Therapy.

The bold values indicate significant P values.

**Figure 4:**
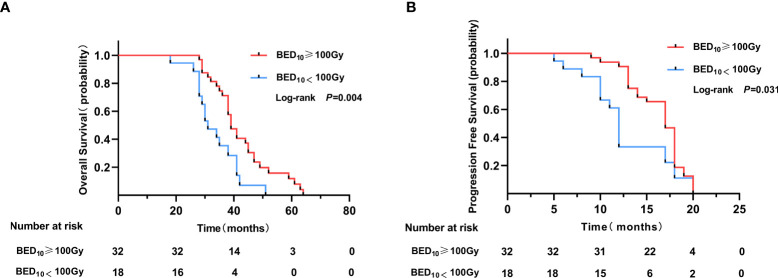
Overall survival and progression free survival of primary tumors treated with BED_10_ ≥100 Gy **(A)** or BED_10_ <100 Gy **(B)** in the TKIs + LCRT group.

In the TKIs + LCRT group, clinical factors for primary tumor, BED ≥100 Gy, were associated with better OS and PFS in univariate analyses (p = 0.004 and p = 0.031, respectively) (**Figure 4**). However, multivariate analysis revealed that the difference was not significant (p = 0.19 and p = 0.61, respectively).

### Toxicity

For non-hematological adverse effects during EGFR-TKIs therapy, the most common events were skin rash, diarrhea, fatigue, nausea, vomiting, increased ALT levels, and pneumonitis. Most of the patients presented with grade 1–2 toxicities. Incidences of >grade 3 skin rash was 4.9% (n = 6). For radiotherapy-related acute side effects, most of the patients exhibited grade 1–2 toxicity, with very few patients exhibiting grade 3 toxicities. The incidence of grade 1–2 radiation pneumonitis in the TKIs + LCRT group was 26% (n = 13), while grade 3 pneumonia (n = 3, 6%) was only found in COPD patients. Two patients treated with head gamma knife (22 Gy/1 f) were subjected to asymptomatic radiation necrosis.

## Discussion

Clinical incidence for metastatic NSCLC among NSCLC patients is approximately 50%. Metastatic NSCLC is more common in older patients than in younger patients (12). This is because, in elderly patients, the disease is at a more advanced stage at the time of diagnosis. In this study, the median age for the 1,857 metastatic NSCLC patients at the time of diagnosis was 63.5 years. Approximately 20–50% of advanced NSCLC patients were oligometastatic NSCLC (13). There is no universally accepted definition of oligometastases. However, recent studies have described oligometastases as 3–5 metastases (8–10). PET-CT has an excellent sensitivity in detecting distant and occult metastases. Detection of oligometastatic disease can be more accurate when combined with brain MRI (14, 15). Approximately, 70% of patients (n = 85) received PET-CT examinations. This enabled precise determination of clinical stages of oligometastatic NSCLC. Few clinical trials have included elderly patients with advanced tumors. Therefore, it can be difficult to choose appropriate therapeutic approaches for elderly patients with advanced tumors, leading to insufficient and inappropriate treatment (16). Apart from surgery and chemotherapy, radiotherapy is one of the most important methods for cancer treatment. Very elderly patients have been shown to tolerate radiotherapy, both in definitive and palliative settings (17, 18). Schmid et al. (19) reported that adoption of novel treatment approaches for the elderly population is lagging. This study was retrospective in nature, therefore, based on clinical records, we concluded that elderly patients treated with TKIs alone at the time did not receive radiation therapy because of: fear and anxiety regarding radiotherapy; lack of radiotherapy information resources; concerns regarding acute and long-term side effects; lack of symptoms of pain and a low willingness for radiation treatment.

NSCLC patients with EGFR activating mutations and who are treated with TKIs exhibit longer PFS and higher response rates, compared to those with EGFR mutation-negative tumors treated with conventional chemotherapies (20, 21). Elderly patients and patients with poor PS have been shown to have similar clinical benefits as younger and fitter patients, therefore, they should be offered targeted treatments in case of oncogenic driver alterations (22). However, within a year, majority of the patients eventually progress, primarily due to acquired resistance in the EGFR kinase domain. Elderly patients treated with TKIs and LCRT exhibited longer mPFS and mOS, relative to those treated with TKIs alone (17 vs.12 months, p <0.001; 38 vs. 29 months, p <0.001, respectively). Multivariate analysis showed that LCRT constitutes one of the independent favorable prognostic factors for PFS (HR = 0.32, 95%CI: 0.20–0.51, p <0.001), and OS (HR = 0.41, 95%CI: 0.27–0.63, p <0.001). These results are in accordance with recent studies, which reported that among 231 patients with EGFR mutant stage IV NSCLC, mPFS and mOS were significantly longer for the local consolidation therapy (surgery or radiotherapy) plus TKIs group than the TKIs monotherapy group (23).

Traditionally, local radiotherapy is considered to be a palliative care for elderly patients with advanced metastatic tumors. However, treatment goals and strategies have changed from palliative care to improvement of PFS and OS. This is attributed to the introduction of the concept of oligometastatic disease. A retrospective study found that in EGFR mutant oligometastatic NSCLC patients, administration of EGFR-TKIs with local consolidation radiation therapy resulted in significantly longer mPFS, compared to TKIs monotherapy (36 vs.14 months, p = 0.0024) (24). A phase 2 randomized trial involving 29 patients reported significant benefits in PFS with addition of consolidative radiotherapy to maintenance chemotherapy for patients with oligometastatic NSCLC (9.7 vs. 3.5 months) (25). Studies have reported that patients with EGFR-positive NSCLC are more likely to develop brain metastases (26, 27). In this study, 50.8% of the patients (n = 62) had brain metastases. Survival analysis did not reveal statistically significant differences in survival probabilities between patients with or without brain metastases (p = 0.342). However, in the subgroup with brain metastases, OS was significantly improved by LCRT for brain metastases (38 vs. 25 months, HR = 0.38, 95%CI: 0.21–0.69, p = 0.001).

Significant comorbidities can limit life expectancy, decreasing potential survival benefits from cancer treatment (28, 29). In this research, 80.3% of the elderly patients (n = 98) had underlying diseases. Only three patients died of acute myocardial infarction, with no tumor progression. Survival analysis showed that there was no evident effect of comorbidities on prognostic outcomes (HR = 1.23, 95%CI: 0.83–1.82, p = 0.300). This may be relevant in relation to how underlying diseases are controlled. In oligometastatic NSCLC, a good quality of life, a small number of metastatic sites, a small primary lung mass size and local consolidation therapy are favorable prognostic factors (30, 31). In this study, good ECOG PS (HR = 0.54, 95%CI: 0.34–0.85, p = 0.007) was established to be an independent favorable prognostic factor for OS and PFS, consistent with a previous study by Sheu et al. (32). A good physical score indicated that the quality of life was satisfactory, nutritional intake was sufficient, and body immune function played a partial role. This ensured treatment continuity as the disease progressed. Moreover, we found that one to two metastases (HR = 0.57, 95%CI: 0.39–0.83, p = 0.004) were independent prognostic factors for better PFS. This implies that patients with a small number of metastatic sites have a relatively low probability for widespread metastatic disease from localized diffusion. Survival analysis revealed that LCRT to primary tumor and all oligometastatic sites is an independent favorable prognostic factor for OS and PFS, in tandem with findings from previous studies (8, 33). A study reported a high local control rate in hypofractionated radiotherapy (BED_10_ ≥100 Gy) lesions (34). In this study, 64% (n = 32) of the patients in the TKIS + LCRT group were subjected to hypofractionated radiotherapy (BED_10_ ≥100 Gy) for primary tumor. In subgroup survival analysis (TKIs + LCRT group), patients who received BED_10_ ≥100 Gy for primary tumor exhibited significantly better survival benefits in OS (39 vs. 31 months, p = 0.004), and PFS (17 vs. 13 months, p = 0.031). This shows that a high local control rate for primary tumor can translate into a survival benefit for elderly patients with oligometastases. Herrera et al. reported that by performing stereotactic body radiation therapy (SBRT) to the local tumor, the body can activate systemic anti-tumor immune effects, enhancing cellular immunity (35).

SRS (Gamma Knife, CyberKnife, and standard linear accelerator) for the intracranial oligometastatic disease has shown promising results. SBRT, a non-surgical alternative for oligometastatic disease, is associated with low reported toxicity and impact on the quality of life. Compared to conventional radiotherapy, SRS and SBRT have numerous advantages that increase the probability of local control while maintaining the risk of normal tissue toxicity at acceptable levels (36). Cuccia et al. (37) reported that 61 elderly patients (median age 82 years) with 90 oligometastases were treated with SBRT with a median BED10 100 Gy (range, 48–180 Gy). Local control rates at 1- and 2-years were 98.8 and 88.2%, respectively, and there were no grade 2 or higher adverse events. In this study, most patients received SBRT, stereotactic radiosurgery (SRS), or hypofractionated radiotherapy, while some patients received conventional fractionated radiotherapy. When the primary tumor was close to the esophagus or in combination with severe chronic obstructive pulmonary disease, the primary tumor was treated with conventional fractionated radiotherapy (70 Gy in 30 or 35 fractions). Most of the patients experienced grade 1–2 radiation-related esophagitis, while none of the patients developed grade 3–4 esophagitis. Vertebral metastases were treated with 30 Gy in 10 fractions or 40 Gy in 20 fractions, since the planning target volume (PTV) encompasses the entire vertebra. Meanwhile, metastasis of superficial lymph nodes was treated with conventional fractionated radiotherapy to reduce incidences of severe radiation dermatitis.

In the FLAURA study, osimertinib showed superior clinical outcomes, compared to standard EGFR-TKIs in EGFR-mutated NSCLC (38). Osimertinib became a preferred first-line treatment modality in previously untreated advanced NSCLC harboring EGFR activating mutations. Zeng et al. (39) reported that 108 patients treated with osimertinib later developed oligometastatic disease. They also reported that for 14 patients who received local consolidation therapy, mPFS was significantly longer than for the osimertinib alone group (NR vs. 12.8 months, p = 0.01), and were independently associated with prolonged PFS (HR = 0.29, 95%CI: 0.12–0.68, p = 0.004). In this study, most of the patients (77.9%) received gefitinib as first-line treatment, while only 7 patients received osimertinib as subsequent treatment. This may be attributed to clinical guidelines, heavy cost of treatment, individual treatment strategies, and family treatment wishes. Approximately 50% of patients in both groups did not receive any second line treatment, including next-generation TKIs. This was attributed to reluctance to accept intensive chemotherapy and cost implications. For instance, before November 2018, the cost of osimertinib was mostly met by households through out-of-pocket expenditure. However, with the cost of osimertinib being covered by health insurance, more EGFR-mutated NSCLC patients received osimertinib as the first-line treatment. In this study, after initial first-line TKIs treatment, 72 patients in the TKIs alone group were not subjected to any radiotherapy in case there was no progression. Fourteen (19.4%) patients with symptomatic brain metastases received palliative radiotherapy (WBRT 40 Gy in 20 fractions or WBRT 30 Gy in 10 fractions). Twelve (16.7%) patients received local radiotherapy for palliation of painful bone metastases (30 Gy in 10 fractions or 40 Gy in 20 fractions).

This study had several limitations. i. The sample size was small, making it difficult to detect statistical differences in some subgroups. ii. It is a retrospective study, and the patients were from two centers. Patients from the Beijing Geriatric Hospital were older than those from the Air Force General Hospital (median age 73 vs. 68 years). Moreover, patients from the Beijing Geriatric Hospital had more comorbidities, compared to those from the Air Force General Hospital. Therefore, the center spends more time dealing with comorbidities before performing LCRT. Segmentation plans and doses of LCRT were also different in the two centers. Aggressive treatment approaches were performed at the Air Force General Hospital. iii. Elderly patients were not evaluated by comprehensive geriatric assessment (CGA) and did not further clarify which type of oligometastatic NSCLC benefited more from LCRT.

## Conclusion

LCRT for primary tumor and all oligometastatic sites in elderly patients with oligometastatic NSCLC harboring EGFR activating mutations during first-line EGFR-TKIs treatment may improve their survival outcomes with tolerable toxicity. This is a potential treatment approach for elderly patients with oligometastatic disease.

## Data Availability Statement

The datasets used and/or analyzed during the current study are available from the corresponding author on reasonable request. Requests to access these datasets should be directed to: xiaodaigua903@163.com.

## Ethics Statement

The studies involving human participants were reviewed and approved by The Ethics Committee of Beijing Geriatric Hospital.

## Author Contributions

Conception and design: YW, and XH. Administrative support: JZ. Provision of study materials or patients: CL and YW. Data collection and assembly: XH, HL, and HP. Data analysis and interpretation: XH, HL, and XK. Manuscript writing: All authors. All authors contributed to the article and approved the submitted version.

## Funding

This work was supported by Beijing Geriatric Hospital Special Fund for Geriatrics Research (2020bjlnyy-q-1).

## Conflict of Interest

The authors declare that the research was conducted in the absence of any commercial or financial relationships that could be construed as a potential conflict of interest.

## Publisher’s Note

All claims expressed in this article are solely those of the authors and do not necessarily represent those of their affiliated organizations, or those of the publisher, the editors and the reviewers. Any product that may be evaluated in this article, or claim that may be made by its manufacturer, is not guaranteed or endorsed by the publisher.
